# Optimal ICG dosage of preoperative colonoscopic tattooing for fluorescence-guided laparoscopic colorectal surgery

**DOI:** 10.1007/s00464-021-08382-5

**Published:** 2021-02-26

**Authors:** Hong-min Ahn, Gyung Mo Son, In Young Lee, Dong-Hoon Shin, Tae Kyun Kim, Su Bum Park, Hyung Wook Kim

**Affiliations:** 1 Department of Surgery, Pusan National University Yangsan Hospital, 50612, 20, Geumo-ro, Mulgeum-eup, Yangsan-si, Gyeongsangnam-do, Yangsan, Korea; 2 Research Institute for Convergence of Biomedical Science and Technology, Pusan National University Yangsan Hospital, Yangsan, Korea; 3 Department of Pathology, School of Medicine, Pusan National University, Yangsan, Korea; 4 Department of Anesthesia and Pain Medicine, Pusan National University, Yangsan, Korea; 5 Department of Internal Medicine, School of Medicine, Pusan National University, Yangsan, Korea; 6 Medical Research Center, School of Medicine, Pusan National University, Yangsan, Korea

**Keywords:** Tattooing, Fluorescent dyes, Fluorescein angiography, Colonoscopy, Laparoscopy, Colorectal neoplasm

## Abstract

**Background:**

Indocyanine green (ICG) is a multifunctional dye used in tumor localization, tissue perfusion, and lymph node (LN) mapping during fluorescence-guided laparoscopic colorectal surgery.

**Purpose:**

This study aimed to establish the optimal protocol for preoperative endoscopic submucosal ICG injection to perform fluorescence lymph node mapping (FLNM), along with undisturbed fluorescent tumor localization and ICG angiography during a single surgery.

**Methods:**

Colorectal cancer patients (*n* = 192) were enrolled from May 2017 to December 2019. Colonoscopic submucosal ICG injection was performed 12 to 18 h before surgery. ICG injection protocols were modified based on the total injected ICG (mg) and tattooing site number. The concentrations of ICG were gradually decreased from the standard dose (2.5 mg/ml) to the minimum dose (0.2 mg/ml). Successful FLNM (FLNM-s) was defined as distinct fluorescent LNs observed under NIR camera. The patient’s age, sex, body mass index (BMI), stage, cancer location, obstruction, and laboratory findings were compared between the FLNM-s and failed FLNM (FLNM-f) groups to identify clinical and pathological factors that affect FLNM.

**Results:**

In the ICG dose section of 0.5 to 1 mg, the success rate was highest within all functions including FLNM, fluorescent tumor localization, and ICG angiography. FLNM-s was related to ICG dose (0.5–1 mg), multiple submucosal injections, location of cancer, camera light source, and lower BMI. In the multivariate analysis, camera light source, non-obesity, and multiple injections were independent factors for FLNM-s). The mean total number of harvested LNs was significantly higher in the FLNM-s group than that in the FLNM-f group (*p* < 0.001). The number of metastatic lymph nodes was comparable between the two groups (*p* = 0.859).

**Conclusions:**

Preoperative, endoscopic submucosal ICG injection with dose range 0.5 to 1 mg would be optimal protocol for multifunctional ICG applications during fluorescence-guided laparoscopic colorectal surgery.

Colorectal cancer was the third most commonly diagnosed cancer and the second most common cause of cancer-related death. With the help of advanced technology, minimally invasive surgery using laparoscopic and robotic surgical systems is performed increasingly for colorectal cancer. However, recognizing the location of early-stage tumors using laparoscopic or robotic devices has become challenging [1]. To overcome the absence of tactile feedback, many intraoperative navigation methods have been introduced, including intraoperative ultrasound, gamma probes, and preoperative endoscopic tumor marking, referred to as “tattooing” [1, 2]. Indocyanine green (ICG) is a tricarbocyanine iodide dye molecule that is amphiphilic, relatively non-toxic, and dark blue pigmented substance that can be used as one of good tattooing agents. ICG is a useful preoperative tattooing agent that fluoresces under near-infrared (NIR) light with a maximum peak wavelength of approximately 830 nm [2, 3]. With the use of fluorescence imaging systems, the different dilutions of the ICG can be used to distinguish the colon from the mesentery and visceral fat tissue under NIR illumination [4, 5]. Tattooing with ICG makes it easy to localize the tumor under NIR system even with the extremely low injection dosage [6].

In colorectal cancer surgery, ICG has multiple applications such as fluorescent tumor localization, intraoperative angiography, and fluorescence lymph node mapping (FLNM) [3, 7]. Intravenously injected ICG rapidly binds to plasma proteins and remains within the bloodstream [1, 3]. This allows the operator to perform intraoperative ICG angiography to find favorable perfusion segment before the colon transection. By optimizing the perfusion at the anastomosis, ischemic complications can be prevented [8]. On the other hand, when ICG is injected into subserosal, submucosal, or subcutaneous layers, it spreads through the lymphatic system and binds to macrophages within lymph nodes (LN). [9]. Lymphography with ICG is suitable because of slow interstitial fluid reabsorption and the backflow of lymphatic drainage from the distal perivascular space [9]. Many studies have tried to identify the lymphatic pathways involved in cancer using preoperatively injected ICG under NIR light, and this approach is proved as good modality for visualize the lymphatic pathways of colorectal cancer [10, 11].

Tattooing protocols, such as the injection method and ICG dosage, vary between facilities and surgeons. In addition, because of its many applications, previous studies focused on each of its properties one at a time on the use of ICG in colorectal surgery. Even though all of its functions, including tumor localization, angiography, and lymphography, can be considered as essential steps in a surgical procedure, conventional ICG tattooing protocol for tumor localization was unsuitable if the aim was to take advantage of all the surgical applications of ICG at once. This study, thus, aimed to establish the optimal protocol for preoperative endoscopic submucosal ICG injection to obtain FLNM with both fluorescent tumor localization and ICG angiography within a single surgery. Furthermore, we evaluated the clinical and technical factors that may affect FLNM on colorectal cancer surgery.

## Methods

### Patients

This prospective study included 192 patients who underwent laparoscopic surgery for colorectal cancer from May 2017 to December 2019 at Pusan National University Yangsan Hospital. The inclusion criteria were patients aged 19–80 years who had colon or rectal cancer and underwent laparoscopic colorectal surgery, with or without diverting ileostomy according to the location of the cancer. The exclusion criteria were hemodynamic instability, emergent surgery, and pregnancy. All patients enrolled in the study were found to have no history of allergies or adverse effects to either the contrast agent used for computed tomography (CT) or the iodine-containing drugs used to reduce the risk of cross-reaction. This study was conducted after receiving the approval of the Institutional Review Board (IRB No. 05-2018-182) of the Pusan National University Yangsan Hospital. Written informed consent was obtained from all patients included in this study.

### Preoperative colonoscopy

**Fig. 1:**
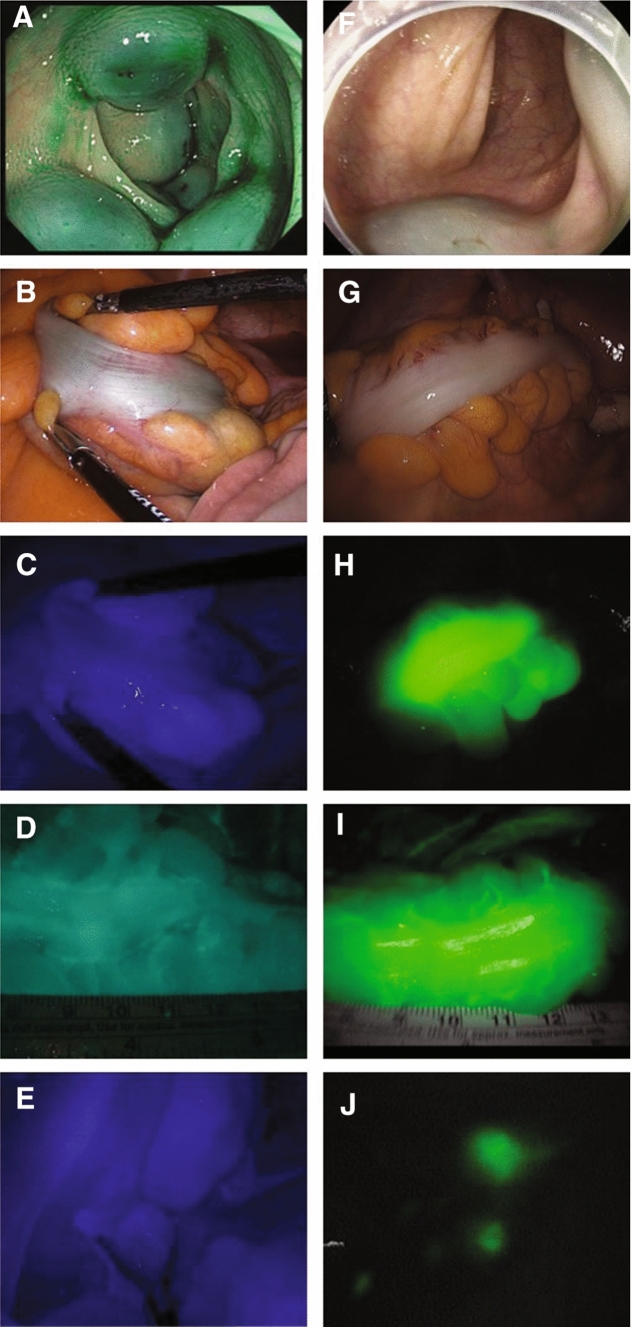
ICG application in laparoscopic colorectal surgery. Conventional dosage of ICG (total injected dose of 25 mg at 2.5 mg/ml, injected in doses of 2.5 ml) was injected into submucosa of the four quadrants surrounding the cancer (**A**). Tattooing with conventional protocol makes the gross localization possible using the naked eye (**B**); however, under NIR illumination, it is harder to distinguish the cancer from the surrounding tissues (**C**). ICG angiography was disturbed by the stained surrounding tissues following conventional tattooing (**D**). FLNM failed because of the influence of the stained mesentery following the use of a high dose of tattooing agent (**E**). Endoscopic tattooing with diluted ICG is suggested as the optimal protocol (total injected dose of 0.5 mg at 0.25 mg/ml, injected in doses of 1 ml at two separate sites) (**F**). The gross localization of the tumor was challenging (**G**); a definite separation between tumor and surroundings was seen using the NIR system (**H**). Successful ICG angiography was performed (**I**), and FLNM was well established under NIR illumination (**J**)

All patients were hospitalized two days before their surgery and received mechanical bowel preparations with a bottle of Picosolution (Picosolution Soln 170 ml/btl, Korea McNulty’s co Ltd) and 2 L of water at 6 PM, followed by taking 2 tablets of Bisacodyl (Bisacodyl 5 mg/tab, Sinil Pharm Ltd) before sleep. They underwent endoscopic submucosal tattooing 12 to 18 h before surgery. ICG (DIAGNOGREEN INJ. 25 mg, Daiichi Sankyo, Tokyo, Japan) was directly injected into the submucosa of the colon or rectum that was slightly distal to the tumor (Fig. 1). The preoperative nil per os (NPO) condition was maintained until the surgery.

During the study period, we had varied the colonoscopic tattooing protocols based on the ICG dilution concentration (mg/ml) and number of injection sites (single or multiple) to obtain optimal ICG fluorescence utilization. At the initial period of study, 25 mg of ICG solution (10 ml) was injected at the quadrant of perpendicular plane to the long axis of tumor. In order to improve tumor localization, intraoperative angiography, and FLNM, simultaneously, the dilution concentrations of ICG were gradually decreased. The total amounts of injected ICG were divided into five categories: > 12 mg, 1–12 mg, 1–0.5 mg, 0.5–0.3 mg, and < 0.3 mg. The submucosal ICG injection sites were also reduced to single point at peritumoral area (Fig. 1). During the course of study, we had changed ICG injection to two or more sites to prevent technically inadequate submucosal injection of diluted ICG. It has been recommended to inject dying agents at least 2 cm distal to the lesion [12]. This could help secure the distal resection margin and prevent the spread of cancer through the injection needle. In order to visualize the lymphatic pathways of the tumor more accurately, ICG injection sites were placed as close as possible to the distal margin of the tumor.

### Fluorescent tumor localization

The tumor localization was evaluated after the laparoscopic NIR camera had been introduced through a transumbilical trocar. We used three different kinds of laparoscopic fluorescence imaging systems during the study period: Stryker (1588 AIM camera system, Stryker, USA), Storz (IMAGE1 S™, Karl Storz, Germany), and Olympus (CLV-S200-IR, Olympus, Japan). AIM camera system uses a Laser as the light source, whereas the other two imaging systems use Xenon lamp. The imaging modality depended on the instrument that was available for use at the time the surgery was being performed.

In white light view of the laparoscopic camera, the gross localization was evaluated. Using the same aspect, the NIR camera mode was changed to ICG image to identify the fluorescent tumor localization (Fig. 1). The tumor localization score were recorded using the 3-point visibility scale developed by Price et al., where score 0 represents not seen, score 1 represents seen with difficulty, and score 2 represents easily seen [13, 14]. We defined failed localization as a score of 0, whereas a score of 1 or 2 was considered successful localization.

### ICG angiography

All patients underwent intraoperative ICG angiography before colon transection. The ICG was prepared by the anesthesiologist after dilution in 10 ml of distilled water. Body weight-adjusted doses of ICG (0.2–0.25 mg/kg) were injected slowly for 10 s. Colonic perfusion was monitored for 2 min after the ICG injection. We obtained the fluorescence perfusion video using the ICG mode of the laparoscopic NIR camera and perform the quantitative analysis methods introduced in our previous study [8]. When quantitative analysis was possible using recorded ICG perfusion video, those cases were considered as “successful ICG angiography.” On the other hand, excessive preoperative submucosal ICG injection causes the disturbance of the dye-surrounded tissues and ICG perfusion analysis was impossible, so those cases were classified as “failed ICG angiography.”

### Fluorescence lymph node mapping

**Fig. 2:**
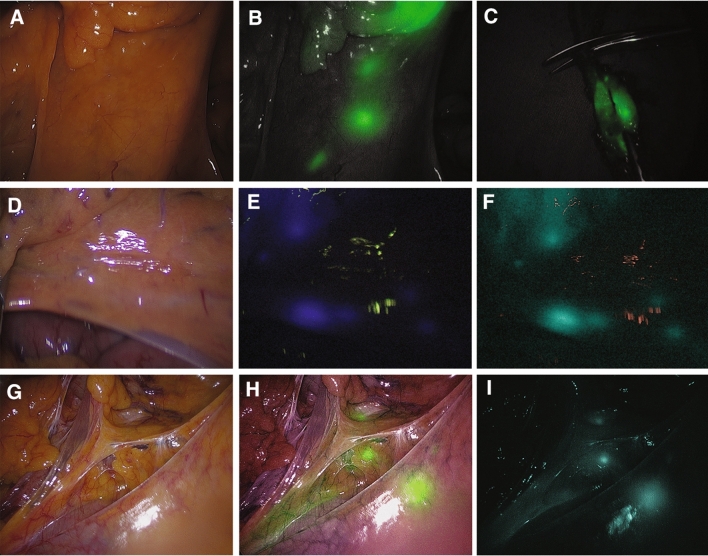
Fluorescence lymph node mapping (FLNM). Three different imaging systems were used during the study period: Stryker (1588 AIM camera system) (**A**–**C**), Storz (IMAGE1 S™) (**D**–**F**), and Olympus (CLV-S200-IR) (**G**–**I**). (**A**), (**D**), and (**G**) show the mesentery of the colon under white light. Green fluorescence-colored LNs are seen using the Endoscopic Near-Infrared Visualization (ENV) mode of the Stryker (**B**). Isolating the ICG-dyed LN from the specimen, which is fully resected from the surgical field (**c**). Blue-colored LNs are seen under the conventional ICG mode (**E**) and green-colored under the Spectra mode (**F**) of the Storz. Partial white light and infrared (IR) light at the same time lead to green-colored LNs overlapping with original view as seen in the narrow-band imaging (NBI) mode (**H**) of the Olympus; green-colored LNs are seen in the IR mode (**I**)

FLNM were explored before initial LN dissection (Fig. 2). By using the ICG image mode, definite circular or crescent-shaped fluorescent LNs were considered as successful FLNM (FLNM-s). On the other hand, cases with either no fluorescent LNs or non-distinguishable fluorescent LNs were classified as failed FLNM (FLNM-f). Radical D3 lymphadenectomy was performed in all patients. When fluorescence LNs were observed outside of the D3 area, such as paraaortic nodes, they were defined as D4 LNs, and LN dissection was extended. After radical LN dissection was completed, the absence of any residual fluorescent LNs was confirmed by using ICG mode of laparoscopic NIR camera.

After the specimen was extracted through the transumbilical mini-laparotomy, we transected proximal margin of bowel and prepared proximal colon for end-to-end anastomosis. By using the laparoscopic NIR camera, fluorescence LNs on the D3 territory were harvested carefully from the specimen, which were identified as ICG-labeled LNs for a separate pathological examination. To avoid cancer cell spillage in the surgical field, the operator did not isolate fluorescent pericolic or intermediate LNs close to the primary tumor, individually. With the FLNM-s group, the remaining apical LN contained tissues were also separated and sent to additional pathologic evaluation for the non-fluorescence LNs that might remain after extracting fluorescence LNs. In the FLNM-f group, apical LN tissues containing D3 LNs were dissected by a surgeon and sent to pathological evaluation for metastasis.

The pathologist evaluated LNs from each labeled tissues and isolated the pericolic LN buried deep in the mesocolon or mesorectum. Pathologic evaluations were performed separately on labeled fluorescence D3 LNs and non-fluorescence D3 LNs. Hematoxylin & Eosin (H&E) staining was used to assess metastatic LNs. The numbers of harvested LNs and metastatic LNs were counted. The harvested LNs were divided into pericolic and D3 area. When LN was not found by H&E staining in the fluorescence D3 tissue, it was treated as no harvested LN. The non-fluorescence D3 LNs were explored for the residual and metastatic LNs. ICG-labeled D4 LNs were also evaluated in the same manner.

### Statistical analysis

Chi-square test or Fisher’s exact test was applied to compare the categorical variables in a univariate analysis. Multivariate analysis was performed with binary logistic regression model using a forward condition analysis. The covariance input criterion was less than 0.1 and elimination criterion was less than 0.05. The correlation between the number of harvested LNs and metastatic LNs was analyzed using the Mann–Whitney U test. All statistical analyses were performed using the Statistical Package for the Social Science (SPSS) version 23.0 for Windows (SPSS/IBM, Chicago, IL, USA). All results with a p-value of less than 0.05 were considered significant.

## Results

**Fig. 3:**
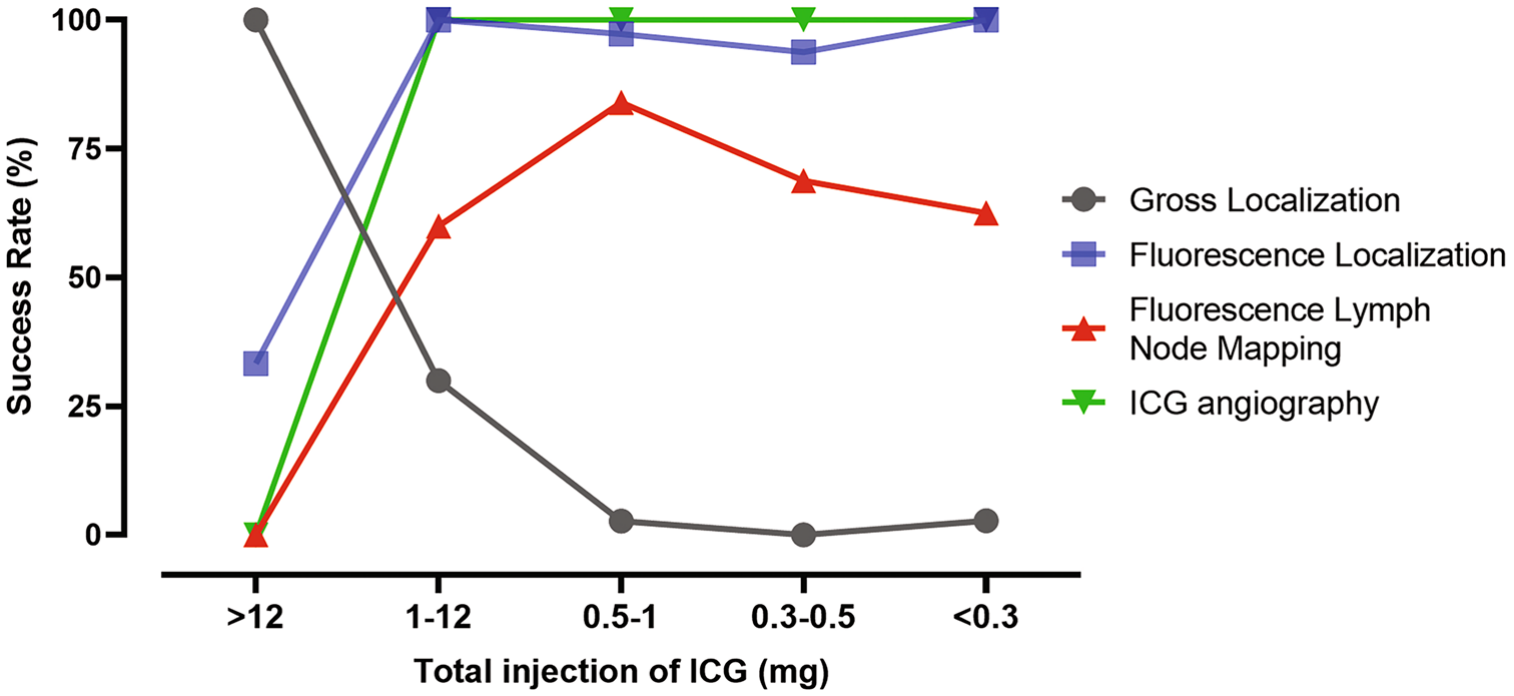
Optimal ICG tattooing protocol. Within an injection dosage of 0.5–1.0 mg, the optimal protocol achieves successful Fluorescence lymph node mapping (FLNM) along with fluorescence localization and good ICG angiography during a single surgery

Characteristics of patients (*n* = 192) are shown in Table 1. With decreased dilution concentration of ICG, the success rate of gross localization under white light gradually decreased. In laparoscopic NIR camera, the dilution allowed the fluorescent tumor localization to be easily distinguished from its surroundings. The success rate of ICG angiography abruptly increased without hindrance of fluorescent tumor localization after using a small amount of submucosal ICG injection. Interestingly, the success rate of FLNM was highest when 0.5 to 1 mg of ICG was injected (84.0%), and this rate decreased when less than 0.5 mg of ICG was injected. By integrating these patterns, we were able to assume that between 0.5 mg and 1 mg of ICG is the optimal dosage for preoperative submucosal ICG injection via colonoscopy (Fig. 3). In the statistical analysis, the total injected ICG dosage was significantly related to the success of FLNM (*p* = 0.002) (Table 2).

**Table 1:** Clinical characteristics of colorectal cancer patients who underwent laparoscopic surgery (*n* = 192)

Characteristic	Values
Age (yr)	65.57 ± 9.88
Sex	
Male	119 (62.0%)
Female	73 (38.0%)
BMI (kg/m^2^)	24.11 ± 3.05
< 25	131 (68.2%)
≥ 25	61 (31.8%)
Cancer location	
Ascending colon	26 (13.5%)
Hepatic flexure	16 (8.3%)
Splenic flexure	7 (3.6%)
Descending colon	3 (1.6%)
Sigmoid colon	66 (34.4%)
Rectum	74 (38.5%)
T stage	
T0	9 (4.7%)
T1	64 (33.3%)
T2	28 (14.6%)
T3	78 (40.6%)
T4	13 (6.8%)
N stage	
N0	139 (72.4%)
N1	42 (21.9%)
N2	11 (5.7%)
Tumor size (cm)	3.46 ± 2.32
Preoperative laboratory study	
CEA (ng/ml)	5.45 ± 20.21
Albumin (g/dl)	4.24 ± 0.38
Image system	
Stryker (1588 AIM camera system)	148 (77.1%)
Storz (IMAGE1 S™)	38 (19.8%)
Olympus (CLV-S200-IR)	6 (3.1%)
Fluorescence lymph node mapping	
Success	136 (70.8%)
Fail	56 (29.2%)
Injected ICG dosage (mg)	
> 12	3 (1.6%)
1–12	10 (5.2%)
0.5–1	75 (39.1%)
0.3–0.5	32 (16.7%)
< 0.3	72 (37.5%)

*BMI* body mass index, *CEA* carcinoembryonic antigen, *ICG* Indocyanine green

**Table 2:** Univariate and multivariate analysis of clinical factors associated with the success of FLNM

	Univariate analysis			Multivariate analysis		
	FLNM-f (*n* = 56)	FLNM-s (*n* = 136)	*P* value	OR	95% C.I	*P* value
Age (yr)			0.857			
< 70	37 (66.1%)	88 (64.7%)		–	–	–
≥ 70	19 (33.9%)	48 (35.3%)				
Sex			0.817			
Male	34 (60.7%)	85 (62.5%)		–	–	–
Female	22 (39.3%)	51 (37.5%)				
BMI			**0.002**			
< 25	29 (51.8%)	102 (75.0%)		0.307	0.148–0.636	**0.001**
≥ 25	27 (48.2%)	34 (25.0%)				
Cancer location			**0.044**			
Left-sided colon	49 (87.5%)	101 (74.3%)		2.021	0.764–5.347	0.157
Right-sided colon	7 (12.5%)	35 (25.7%)				
T stage			0.260			
T1–T2	33 (58.9%)	68 (50.0%)		–	–	–
T3–T4	23 (41.1%)	68 (50.0%)				
N stage			0.847			
N0	40 (71.4%)	99 (72.8%)		–	–	–
N1–2	16 (28.6%)	37 (27.2%)				
Obstruction			0.839			
Negative	46 (82.1%)	110 (80.9%)		–	–	–
Positive	10 (17.9%)	26 (19.1%)				
CEA level (ng/ml)			0.750			
< 5.0	46 (82.1%)	109 (80.1%)		–	–	–
≥ 5.0	10 (17.9%)	27 (19.9%)				
Albumin level (g/dl)			0.673			
≥ 3.5	55 (98.2%)	131 (96.3%)		–	–	–
< 3.5	1 (1.8%)	5 (3.7%)				
ICG tattooing dosage (mg)			**0.002**			
≥ 1.0	7 (12.5%)	6 (4.4%)		1.579	0.273–69.124	0.610
0.5–1.0	12 (21.4%)	63 (46.3%)		1	–	–
< 0.5	37 (66.1%)	67 (49.3%)		0.350	0.155–0.793	**0.012**
Tattooing injection site			** < 0.001**			
Single	16 (28.6%)	10 (7.4%)		4.068	0.899–18.403	0.068
Multiple (≥ 2 sites)	40 (71.4%)	126 (92.6%)				
Camera light source			** < 0.001**			
Xenon	24 (42.9%)	20 (14.7%)		2.856	1.056–7.723	**0.039**
Laser	32 (57.1%)	116 (85.3%)				

*FLNM* fluorescence lymph node mapping, *BMI* body mass index, *CEA* carcinoembryonic antigen, *OR* odd ratio, *CI* Confidence interval, *ICG* Indocyanine green

Depending on FLNM success, patients were divided into two groups: FLNM-s (*n* = 136, 70.8%) and FLNM-f (*n* = 56, 29.2%) and compared clinical factors. Non-obese status (BMI < 25) and tumor location were related significantly with the success of FLNM. The right-sided colon, including the cecum to proximal transverse colon, was a preferred factor for FLNM than left-sided colon and rectum (83.3% vs. 74.3%, *p* = 0.044). The number of ICG injection sites was significantly related to FLNM-s. Multiple tattooing had significantly higher successful FLNM rate than single injection (75.9% vs*.* 38.5%, respectively, *p* < 0.001). The different laparoscopic NIR systems (Laser or Xenon lamp) also affected the success of FLNM (*p* < 0.001). In the multivariate analysis, BMI, total injected ICG tattooing dosage, number of injections, and camera light source were statistically relevant to the successful FLNM (Table 2).

Among 136 patients of successful FLNM group, ICG-labeled fluorescence D3 LNs (1050 LNs) were obtained and 10 metastatic LNs (1.0%) were identified in four patients (2.9%). Non-fluorescence D3 LNs (105 LNs) were also obtained in 16 patients and no metastatic LNs were found. There were eleven patients with ICG-labeled D4 LNs (37 LNs), and five metastatic LNs (13.5%) were identified in one patient (9.1%). The percentage of D3 metastatic LNs in FLNM-s group was not statistically different from that of FLNM-f group (Table 3).

**Table 3:** Harvested and metastatic lymph nodes in the FLNM-fail (*n* = 56) and FLNM-success group (*n* = 136)

FLNM-f	Harvested LNs			Metastatic LNs		
(*n* = 56)	*n*	Mean (range)	LN	*n* (%)	Mean (range)	LN (%)
Pericolic LNs	56	18.1 (1–67)	1015	14 (25.0)	4.1 (1–21)	57 (5.6)
D3 LNs	56	4.7 (0–27)	264	2 (3.6)	1 (1)	2 (0.8)
Total LNs	56	22.8 (2–72)	1279	14 (25)	4.2 (1–22)	59 (4.6)

FLNM-s	Harvested LNs			Metastatic LNs		
(*n* = 136)	*n*	mean (range)	LN	*n* (%)	mean (range)	LN (%)
Pericolic LNs	136	25.3 (5–218)	3442	33 (24.3)	2.64 (1–10)	87 (2.6)
D3 Fluorescence LNs	136	7.7 (0–36)	1050	4 (2.9)	2.5 (1–3)	10 (1.0)
D4 Fluorescence LNs	11	3.4 (1–14)	37	1 (9.1)	5 (5)	5 (13.5)
Total LNs	136	33.3 (7–231)	4529	33 (24.3)	4.2 (1–14)	102 (2.3)

*FLNM* fluorescence lymph node mapping, *LN* lymph node

**Fig. 4:**
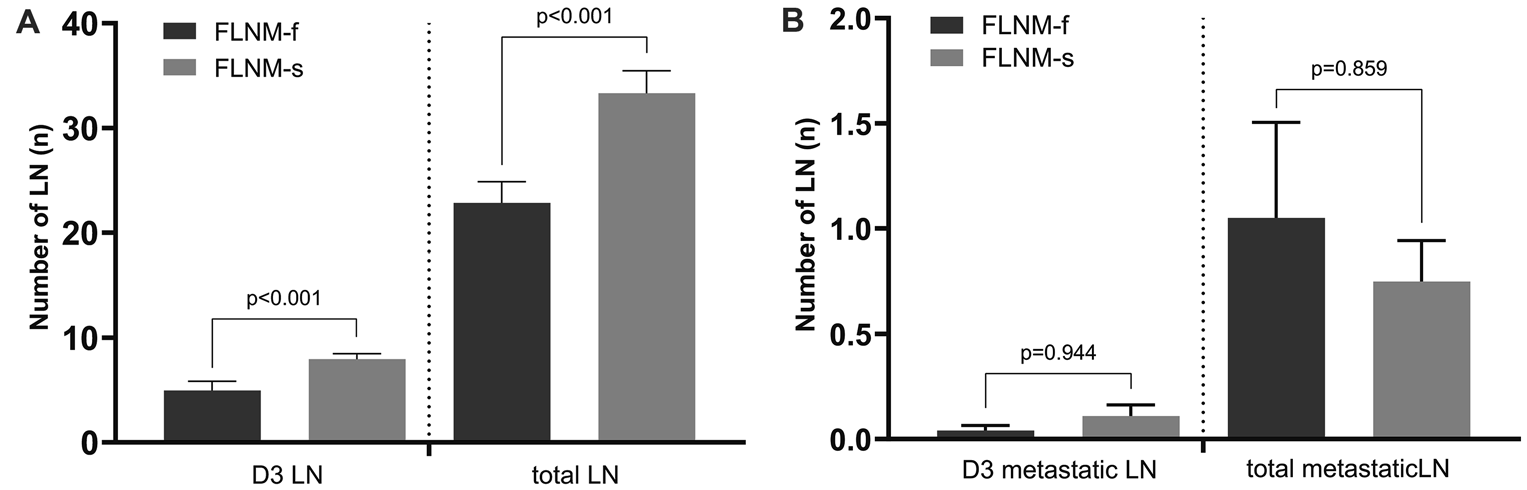
Comparison of the number of harvested lymph nodes between FLNM-s and FLNM-f groups. **A** Total numbers of harvested LNs and D3 LNs were significantly greater in the FLNM group. **B** The number of metastatic LNs was similar in both groups

The number of harvested LNs was compared between the two groups (Fig. 4). The mean total number of harvested LNs was significantly higher in the FLNM-s group than that in the FLNM-f group (*p* < 0.001). The number of metastatic lymph nodes was comparable between the two groups (*p* = 0.859). The number of pericolic LNs was significantly higher in the FLNM-s group than that of the FLNM-f group (25.3 ± 22.2 vs*.* 18.1 ± 12.3, respectively, *p* = 0.024). However, the number of metastatic pericolic LNs was comparable between the FLNM-s and the FLNM-f groups (0.6 ± 1.7 vs*.* 1.0 ± 3.3, respectively, *p* = 0.295).

The number of D3 LNs was also higher statistically in the FLNM-s group than that of FLNM-f group (*p* < 0.001). However, the number of metastatic D3 LNs was not related to the success of FLNM (*p* = 0.944). In the microscopic H&E staining examination of the apical LN tissues, the proportion of no LN was statistically lower in the FLNM-s group than that of FLNM-f group (2.1% vs*.* 10.7%, respectively, *p* = 0.028).

**Fig. 5:**
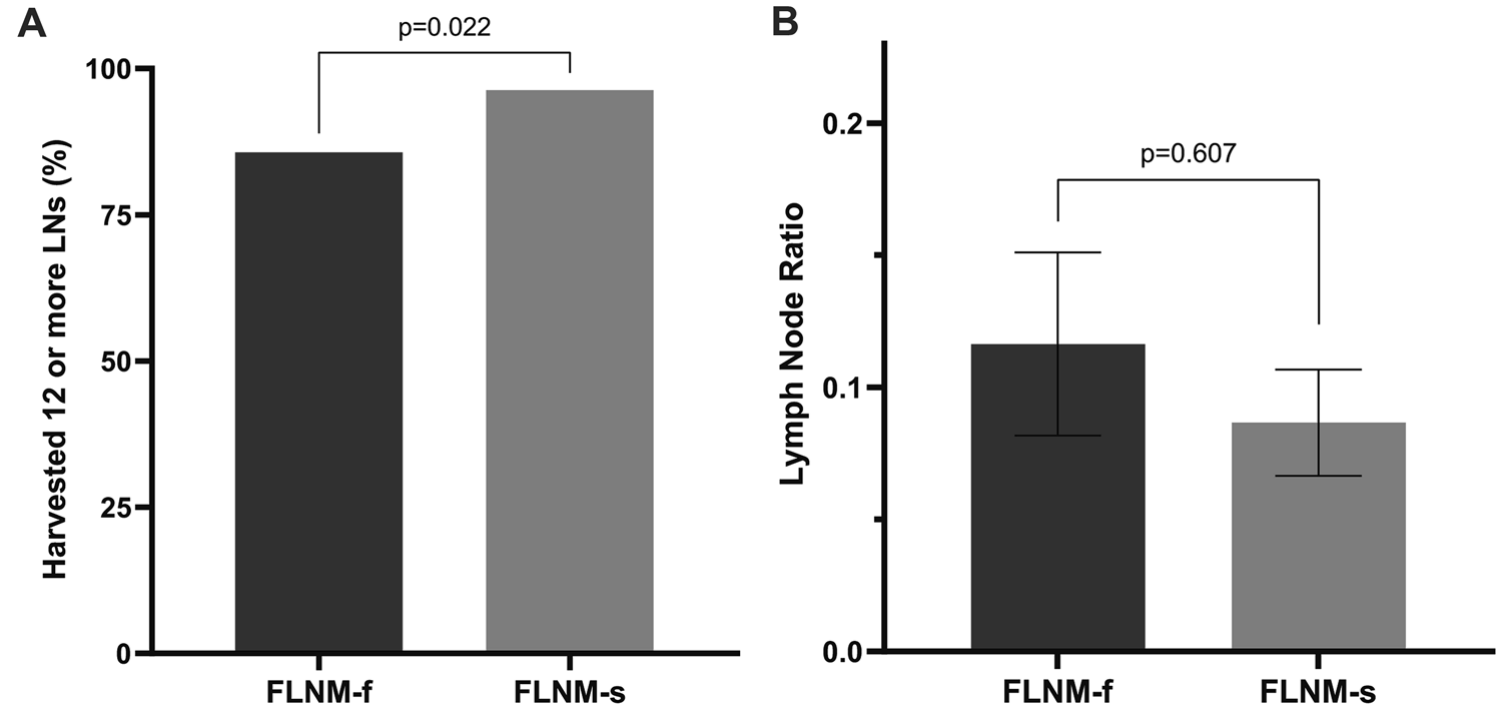
Comparison of oncological aspects under FLNM. **A** In the FLNM-s group, the probability of harvesting 12 or more LNs was approximately four times higher (*p* = 0.022). **B** Lymph node ratio, among stage III patients (*n* = 53), was lower in the FLNM-s group than in the FLNM-f group; however, the difference was not significant (*p* = 0.607)

The sufficiency of LN yields, defined as ≥ 12 harvested LNs, was significantly higher in the successful FLNM group (Fig. 5). The lymph node ratio (LNR) was defined as the number of metastatic LNs divided by total number of retrieved LNs [15, 16]. On the subgroup analysis for stage III disease, the successful FLNM trended to lower LNR; however, the difference was not significant (*p* = 0.607).

## Discussions

All clinical functions of ICG such as tumor localization, ICG angiography, and FLNM play important roles in laparoscopic colorectal surgery [17]. Nevertheless, conventional ICG tattooing protocol had interfered with not only fluorescent tumor localization but also FLNM and intraoperative ICG angiography. Conventional colonoscopic tattooing protocol recommends the injection with high volume and concentration of dye (10 ml solution containing the 25 mg of ICG) as possible in 4 different locations near the tumor [12]. This protocol has been proposed as clear marking on early staged cancer, which can be visualized easily by the operator's naked eyes prior to using NIR camera. However, ICG could spread widely through the colon and surrounding tissue, which made it impossible to localize the tumor under laparoscopic NIR camera.

Thus, we attempted to adjust the dosage and concentration of ICG to obtain an optimized protocol for successful ICG angiography and FLNM as well as fluorescent tumor localization. The initial assumption of this study was that the ICG tattooing injection dosage should be diluted for successful fluorescent tumor localization and ICG angiography. In addition, we have also found that diluting ICG made it possible to map the fluorescent LNs using the laparoscopic NIR camera. Interestingly, extreme dilutions made it more difficult to visualize fluorescent LNs. From this finding, we could suggest the optimal ICG injection protocol: preoperative endoscopic submucosal injection near the lesion at a dose of 0.5–1 mg, which improves success rate of FLNM without the interference of fluorescent tumor localization and ICG angiography. Under this optimal protocol, the injected ICG stays within 1–2 cm distal to the tumor so that it helps determine the safe transection line by definite fluorescent tumor localization. This result can be applied to the basic framework for the standardization protocols for multifunctional ICG applications.

We have narrowed down the clinical factors affecting the success of FLNM to patients’ BMI and the multiple ICG injections technique. It may be an obvious assumption that higher BMI means more visceral fat tissue, which makes it harder to explore the FLNM. The penetration depth of the NIR wave is quite shallow, and in particular, it cannot easily penetrate fat tissue for detecting ICG fluorescence. Especially in Asia, there happened to be many cases with severe visceral obesity with slight elevated BMI, and this explains the importance of BMI as an influencing factor for FLNM.

In addition to the variety of ICG injection dosages, this study showed that injecting at multiple sites helps improve the rate of successful FLNM. At the beginning of the study period, we tried a single injection site with a dilution dose of ICG; however, the success rate of LN mapping was < 40%. Injecting at two or more sites slightly distal to the tumor increased the success rate to 75%. We assumed that multiple injections increased the total ICG dosage, thus allowing easy visualization of LNs using an NIR camera. Another assumption is the reduction in possibility of failure, i.e., if the initial ICG injection failed, subsequent injections may compensate for it. In our study, multiple tattooing had higher success rate of FLNM than single injection.

FLNM in colorectal cancer helps the surgeon optimize the lymphatic pathway to perform more accurate LN dissection [18]. In particular, FLNM can provide excellent visual information to identify dominant lymphatic drainage of the hepatic or splenic flexure cancer with dual lymphatic pathways. Being able to visualize the LNs and lymphatic drainage pathway, the surgeon can dissect LNs much more radically. In addition, a surgeon may change the dissecting plane when a fluorescent LN is outside of the original plane. In this study, we have eliminated all the visible fluorescence LNs during the surgeries by changing the dissection line when the fluorescent appeared outside of the conventional dissection plane. ICG-labeled paraaortic LNs were observed in eleven patients and a total of thirty-seven LNs were removed, of which 5 metastatic LNs were found (13.5%). FLNM helps surgeon perform adequate D3 dissection including extended range of LNs that are easily missed under deep mesentery. However, FLNM also could induce surgeons to dissect further than the conventional D3 area, which may lead to the possibilities of complications. Therefore, FLNM could be applied to evaluate the completeness of LN dissections, but more research should be needed in the field of determining an appropriate oncological LN dissection range, especially for early colon cancer.

In addition, the possibility of metastatic D4 LNs is much higher comparing with fluorescent D3 LNs. It is possible that the percentage of metastatic LNs was higher, since only 11 patients with fluorescent D4 LNs were selectively performed extended radical LN dissection. Nevertheless, we have found an interesting case with extended dissection of fluorescence D4 LNs. From the pathologic results, there were 14 metastatic LNs in pericolic region, but no metastasis was found in the intermediate and apical areas. However, five additional metastatic LNs were found on paraaortic region, which might explain the possibility of skip lymphatic metastasis of rectal cancer. From these results, we were able to acknowledge a careful lesson that the extended radical LN dissection may be a safer surgical strategy, when fluorescent LNs are found outside the D3 LN area, so far, considering the possibility of skip lymphatic metastasis.

Although the total number of harvested LNs was greater in case of successful FLNM, there was no effect on the total number of metastatic LNs. Supporting our results, previous study on comparison of clinical outcomes between ICG-guided laparoscopic right hemicolectomy and conventional surgery has found that harvested LNs were greater but no difference in metastatic LNs by the ICG-guided surgery [19]. On second thought, LN harvest is a function of pathology. Another possible explanation is that pathologist took greater effort on ICG-labeled LNs that surgeon had isolated during surgery. The other possible explanation is that LNs which might have been omitted by the conventional way were harvested with the help of visual aids. Thus, it can be suggested that FLNM helps harvest more LNs than conventional method. Interestingly, in pathologic examination on the H&E staining, a few cases were identified to have no harvested LN on D3 LN basin. In addition, the pathologic failure of D3 LN harvest was significantly lower in the FLNM-s group. Therefore, the successful FLNM could be expected not only to help isolate more LNs, but also for effective pathologic isolation of D3 LNs after radical lymphadenectomy.

From the oncological view, at least 12 LNs should be retrieved to evaluate the pathologic stage of cancer [20]. In addition to the anatomic landmarks, FLNM can be one of the surgical tools that helps in the retrieval of an adequate number of LNs to aid in determining the accurate pathologic stage. Proper mesenteric resection should provide ≥ 12 LNs with or without FLNM. Within conventional method, at least 90% of cases should be performed with harvesting 12 or more LNs. By logic, 86% of FLNM-f group was a notable result in this study. However, among 192 patients, only 13 resulted harvesting less than 12 LNs. This calculates as 6.8%, which makes 93.2% were harvested with adequate number of LNs. Among the 56 patients who had failed FLNM, 8 patients received inadequate LN harvest. This is why the percentage of LN harvest ≥ 12 without ICG was calculated lower than expected.

For further understanding of the oncologic advantages of FLNM, we performed an additional analysis with the subgroup of stage III (N1 or more) colorectal cancer patients to calculate the LNR. Among the prognostic parameters for colorectal cancer, studies of LNR remain ongoing with promising early results. Although studies still need to establish cutoff values for LNR, a lower LNR in stage III colorectal cancer is related to a better prognosis in terms of overall survival and disease-free survival [16, 21]. However, interestingly, some studies showed that LNR was an unsuitable predictor of overall survival in stage III rectal cancer but is still a good prognostic parameter for colon cancer [22]. Since our stage III subgroup included both colon and rectal cancer, it is hard to define whether FLNM is a necessary procedure to establish a good prognostic parameter or not. Despite the statistical significance, we found that the FLNM-s group had a tendency to have lower mean LNR than the FLNM-f group. We hope that this result may lead to further studies of prognostic factors related to FLNM in colorectal cancer surgery.

In this study, the concept of FLNM is not identical to sentinel lymph node (SLN) mapping. The original concept of the SLN can be explained as the hypothetical LNs to which the primary cancer initially drains. Since ICG binds to macrophages within the LNs, FLNM could be considered as promising intraoperative fluorescence image modality for detecting SLNs. In many fields of cancer, especially in breast cancer and malignant melanoma, ICG is used as one of the tools for detecting SLNs so as to minimize surgical procedures and prevent postoperative complications [23]. The value of SLNs has been studied, and it has shown promising results in cervical cancer, endometrial cancer, and gastrointestinal cancer [23, 24]. This is why many previous studies referred to FLNM as the detection of SLN [25, 26]. However, FLNM may not work like SLN mapping on LN-positive patients, because the metastatic LNs are being replaced with cancer cells, which would drain ICG less easily. From the FLNM, the pathway of LN indicates lymphatic flow from the primary tumor; however, within metastatic LNs, the flow was blocked by infiltrated cancer cells. This phenomenon was visually shown by metastatic LNs with insufficient uptake of ICG, which forms none or crescent-shaped fluorescence. In a recent study in Japan, they explored the metastatic LNs under a microscopic NIR camera. Fluorescence did not emit light in the area occupied by cancer cells, and crescent-shaped fluorescence was observed in the node where some of lymphoid tissues still remained [27].

However, in early staged cancer, since ICG submucosal injection can be performed closer to the lesion, it is expected that FLNM, which implies the possibility of occult LN metastasis, will be possible as SLN mapping. In advanced colorectal cancer, FLNM still has limitations in accuracy and reliability on creating a cancer cell spreading pathways. As far as we know, the SLN mapping is also difficult in colorectal cancer, because of the dual lymphatic flow and existence of skipped metastasis. In addition, the range of LN dissection with FLNM is still undetermined in colorectal cancer surgery. In other words, FLNM is still an inadequate modality for detecting the SLN in advanced colorectal cancer. Unfortunately, this study includes not only early staged but also advanced staged cancer. In addition, by consideration of oncologic safety, the fluorescence LNs were not separated from the pericolic LNs, which is likely to be the first drained LN as possible SLN. There are still a lot of questions to be solved, but we believe that FLNM could be one of the important methods for detecting SLN in selected patients. Therefore, we thought that fluorescent LN exploration could focus on the search for dominant lymphatic drainage for early colorectal cancer. Future FLNM studies will serve as the basis for establishing SLN mapping in early colorectal cancer.

There are several limitations in this study. First, this was a small study from a single cohort. Although patients were collected prospectively, sampling with different sizes of ICG dosage groups was unavoidable because protocol update was frequently attempted at the beginning of the study to increase the success rate of fluorescent tumor localization, ICG perfusion, and FLNM. Technically, despite guidance from previous studies, the decision of success and failure mostly depended on subjective measurements. To standardize the optimal protocol of ICG, more objective measurements are needed for large-sized multicenter studies in the future. Second, it may take more effort for pathologists to evaluate ICG-labeled LNs. The labeling may have affected the pathologic examination process of LN sorting, with extra attention being paid to the labeled specimens. This could possibly affect the harvested LN count in the FLNM-s group. Third, all 10 metastatic D3 LNs were extracted from the ICG-labeled LN basin. This is a contrary result to the pathophysiological characteristics that metastatic LNs will not be able to uptake ICG well. The probability of LN involvement in the extracted D3 regional tissue was 97.1% in FLNM-s group, while LN extraction from D3 lymphatic region was 89.1% in FLNM-f group. However, the probability of metastatic LNs among the extracted fluorescence LNs was only 0.8–1.0%. In addition, the metastatic LNs might be included in the ICG-labeled LNs even though ICG was not absorbed. In order to solve this experimental problem, we should plan to re-evaluate the ICG fluorescence of individual metastatic LNs using microscopic NIR camera in the near future. Finally, the clinical and oncological benefit of FLNM has not been yet elucidated in this study. FLNM is based on cancer non-specific ICG staining so that this is unsuitable for finding the metastatic LNs. The range of dissection also is still unclear that FLNM may have benefit to get adequate dissection but unnecessary damage may occur by dissecting too much. Thus, we need to study further to figure out the oncological benefits of FLNM in colorectal surgery. To verify the clinical and oncological benefits, the optimal protocol should be needed at first. This study is focusing on establishing the optimal protocol that FLNM can be well succeeded along with other clinical functions of ICG fluorescence.

In conclusion, we have suggested the optimal protocol of submucosal ICG injection as 0.5–1 mg, 12–18 h preoperatively, so that successful fluorescent tumor localization, ICG angiography, and FLNM under NIR imaging system can be performed within a single surgery. The standardization of multifunctional ICG applications should be established for fluorescence-guided laparoscopic colorectal surgery.
